# Arsenic in Drinking-Water and Risk for Cancer in Denmark

**DOI:** 10.1289/ehp.10623

**Published:** 2007-11-23

**Authors:** Rikke Baastrup, Mette Sørensen, Thomas Balstrøm, Kirsten Frederiksen, Carsten Langtofte Larsen, Anne Tjønneland, Kim Overvad, Ole Raaschou-Nielsen

**Affiliations:** 1 Danish Cancer Society, Institute of Cancer Epidemiology, Copenhagen, Denmark; 2 Department of Geography and Geology, University of Copenhagen, Copenhagen, Denmark; 3 Geological Survey of Denmark and Greenland, Copenhagen, Denmark; 4 Department of Clinical Epidemiology, Aalborg Hospital, Aarhus University Hospital, Aalborg, Denmark

**Keywords:** arsenic, cancer, cohort study, drinking-water, geographic information system

## Abstract

**Background:**

Arsenic is a well-known carcinogen, which is often found in drinking-water. Epidemiologic studies have shown increased cancer risks among individuals exposed to high concentrations of arsenic in drinking-water, whereas studies of the carcinogenic effect of low doses have had inconsistent results.

**Objective:**

Our aim was to determine if exposure to low levels of arsenic in drinking-water in Denmark is associated with an increased risk for cancer.

**Methods:**

The study was based on a prospective Danish cohort of 57,053 persons in the Copenhagen and Aarhus areas. Cancer cases were identified in the Danish Cancer Registry, and the Danish civil registration system was used to trace and geocode residential addresses of the cohort members. We used a geographic information system to link addresses with water supply areas, then estimated individual exposure to arsenic using residential addresses back to 1970. Average exposure for the cohort ranged between 0.05 and 25.3 μg/L (mean = 1.2 μg/L). Cox’s regression models were used to analyze possible relationships between arsenic and cancer.

**Results:**

We found no significant association between exposure to arsenic and risk for cancers of the lung, bladder, liver, kidney, prostate, or colorectum, or melanoma skin cancer; however, the risk for non-melanoma skin cancer decreased with increasing exposure (incidence rate ratio = 0.88/μg/L average exposure; 95% confidence interval, 0.84–0.94). Results adjusted for enrollment area showed no association with non-melanoma skin cancer.

**Conclusions:**

The results indicate that exposure to low doses of arsenic might be associated with a reduced risk for skin cancer.

Arsenic is a ubiquitous element in the environment, where it occurs in both organic and inorganic forms; it can be found in food, water, soil, and airborne particles, and humans are widely exposed through these sources (Tchounwou et al. 2004). Arsenic can cause fatal acute poisoning, and long-term exposure has been associated with various cancers, diabetes, skin disease, chronic cough, and toxic effects in the liver, kidney, cardiovascular system, and the peripheral and central nervous systems (Vahter et al. 2006). Organic arsenic, which is less harmful than the inorganic forms, is most abundant in food, whereas inorganic arsenic compounds are found mainly in aquifers (Abernathy et al. 2003), where they accumulate by natural processes such as weathering, erosion, and biological activity, or eventually from anthropogenic contamination (Smedley and Kinniburgh 2005). Consequently, most health-related problems associated with arsenic are derived from groundwater used for drinking (Farago et al. 1997; Smedley and Kinniburgh 2005).

Epidemiologic studies in Asia (Chen et al. 1986, 1988; Tsuda et al. 1995; Wu et al. 1989) and Latin America (Ferreccio et al. 2000; Hopenhayn-Rich et al. 1996, 1998; Marshall et al. 2007) have shown that high arsenic concentrations (up to several hundred micrograms per liter) in drinking-water are associated with various internal cancers and with cancer of the skin. Some of these studies also provide evidence of a dose–response relation (Chen et al. 1986, 1988; Wu et al. 1989). However, few studies, most of which were conducted in the United States, have addressed the adverse effects of exposure to low doses of arsenic, and their results are inconsistent. Some showed a positive association between relatively low doses of arsenic and cancers of the skin, prostate, and bladder (Knobeloch et al. 2006; Kurttio et al. 1999; Lewis et al. 1999), whereas others showed no such effects (Bates et al. 1995; Karagas et al. 2001; Steinmaus et al. 2003). One study showed a nonsignificant decreasing risk for bladder cancer with increasing exposure to arsenic in the range of 3–60 μg/L (Lamm et al. 2004), and Karagas et al. (2002) found a U-shaped dose–response relation between exposure to arsenic and non-melanoma skin cancer, with a decreased risk at low levels and increased risk at higher levels. The existence of a threshold for the carcinogenic effect of arsenic has been debated, especially in the United States (Abernathy et al. 1996; Schoen et al. 2004), and some studies have suggested an interaction between exposure to arsenic and smoking in the causation of cancers of the lung, bladder and skin (Bates et al. 1995; Ferreccio et al. 2000; Knobeloch et al. 2006; Steinmaus et al. 2003; Tsuda et al. 1995).

Recent animal models for inorganic arsenic carcinogenesis suggest that the carcinogenicity of arsenic involves several mechanisms and co-exposure to other carcinogens (Burns et al. 2004; Cohen et al. 2007; Rossman et al. 2004; Waalkes et al. 2007; Wanibuchi et al. 2004). *In vitro* low concentrations of arsenic protected against oxidative stress and DNA damage (Snow et al. 2005), in accordance with the results of some of the epidemiologic studies (Karagas et al. 2002; Lamm et al. 2004). More studies are needed, however, to evaluate the possible carcinogenic effect of exposure to low concentrations of arsenic. The aim of this large, population-based cohort study was to determine if individual exposure to low levels of arsenic in drinking-water in Denmark is associated with a risk for cancer.

## Materials and Methods

### Study population

The study was based on the prospective Danish cohort Diet, Cancer and Health, which has been described in detail elsewhere (Tjønneland et al. 2007). In brief, 160,725 persons 50–64 years of age and living in one of 23 municipalities in the Copenhagen or Aarhus area were invited to participate. Of these, 57,053 persons (27,178 men and 29,875 women) accepted the invitation and were enrolled between 1993 and 1997. At enrollment, information was collected including on diet, beverages, smoking, education, medical conditions, occupations, reproductive factors, body mass index, and skin reaction to sun. The study “Diet Cancer and Health” has been approved by the relevant Scientific Committees and the Danish Data Protection Agency. Informed consent was obtained from all participants to search information from medical registers including the Danish Cancer Registry.

Since establishment of the Danish Central Population Registry in 1968, all citizens of Denmark have been given a unique personal identification number, which allows accurate linkage among Danish registers. The cohort members were followed up for cancer incidence in the population-based Danish Cancer Registry (Storm et al. 1997) from the time of enrollment until the date of first cancer diagnosis, emigration, death, or 1 August 2003, whichever came first. We included cancers of the lung, bladder, liver, kidney, prostate, female breast, and colorectum, and non-melanoma and melanoma skin cancers. Only first cancers were included, although a case of cancer was included even if it had been preceded by a non-melanoma skin cancer.

Of the 57,053 cohort members, we included 56,378 persons, who filled in the lifestyle questionnaire, reported daily intake of tap water, and had not had a cancer diagnosis before the enrollment.

### Residential histories

**Figure 1:**
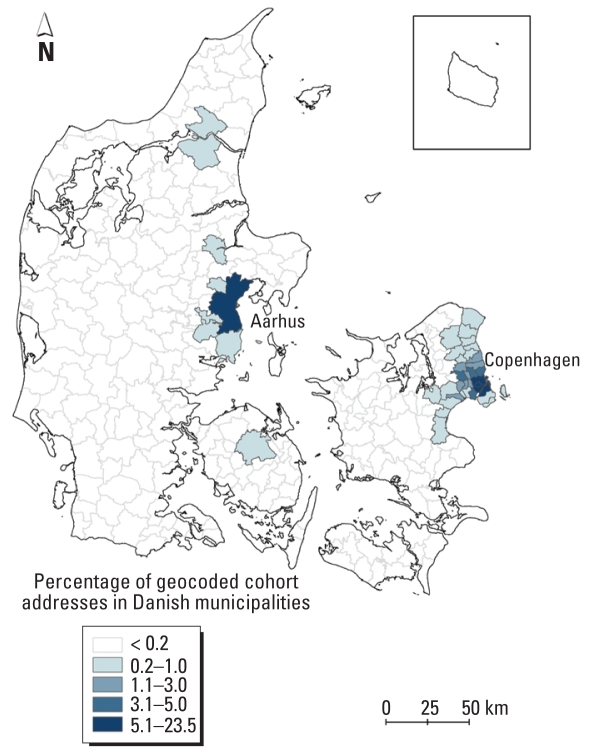
Distribution of geocoded cohort addresses (*n* = 198,758) in 271 Danish municipalities. The proportions are calculated as number of geocoded cohort addresses in each municipality divided by the total number of geocoded cohort addresses.

Using the personal identification numbers of the cohort members, we traced residential histories between 1970 and 2003 by record linkage to the Central Population Registry. With this method, we identified 202,339 unique addresses, each with a unique identification code composed of a municipality code, a road code, and a house number. The date the person had moved to and from the address was noted. The addresses were then linked to a database of all official addresses in Denmark, resulting in geographic coordinates for 198,758 (98%) of the cohort addresses. Subsequently, the addresses were mapped with the ArcGIS 9.1 geographic information system software (Environmental Systems Research Institute, Inc., Redlands, California, USA), and the proportion of addresses in each of the 271 Danish municipalities was calculated in relation to the total number of geocoded cohort addresses (Figure 1).

### Water supply and arsenic measurements

Arsenic concentrations in Danish drinking-water were obtained from a database managed by the Geological Survey of Denmark and Greenland (Thomsen et al. 2004), which contains the results of chemical tests in water utilities in Denmark. Since 2001, it has been compulsory for water utilities to measure arsenic in the drinking-water and to report the results to the database. The spatial locations of the water utilities were determined by their geographic coordinates, which were also registered in the database. We calculated the average arsenic concentration for each water utility on the basis of 4,954 measurements in 2,487 water utilities reported between 1987 and 2004, with most measurements dating from 2002–2004. The average at each water utility was assumed to represent arsenic concentrations throughout the study period 1970–2003. As drilling depth might affect arsenic concentrations and might have changed over time, we collected data on the drilling depth and analyzed the correlation with the arsenic concentration in drinking-water using Spearman’s correlation coefficient.

**Figure 2:**
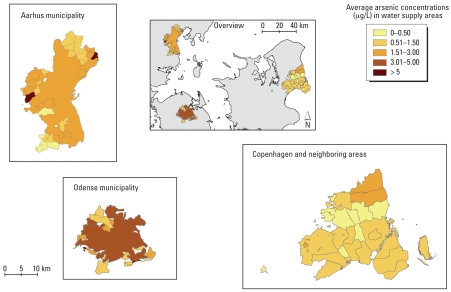
Ninety-four water supply areas classified according to estimated average arsenic concentration (μg/L). These areas cover 84% of the 198,758 geocoded cohort addresses.

To assess the effect of arsenic in drinking-water on the risk for cancer among cohort members, it was essential to link the arsenic concentrations at the water utilities to each address of the cohort members. Therefore, information on the size and spatial location of 94 water supply areas was collected from local authorities and water utilities in 24 municipalities, covering the vast majority of the geocoded cohort addresses. Seventy-one of the collected water supply areas were supplied by only one water utility, whereas the water in 23 areas came from more than one utility. Therefore, we also collected details of the volume of water distributed from water utilities to these 23 areas to calculate water volume-weighted average arsenic concentrations. If, for example, an area received 40% of its water from one utility and 60% from another, the arsenic concentration in the area would be calculated as 0.4 × concentration at utility1 + 0.6 × concentration at utility2. The 94 water supply areas were mapped in ArcGIS 9.1 (Figure 2) and covered 84% of the cohort addresses.

The geocoded cohort addresses, water utilities, and water supply areas with their arsenic concentrations were mapped in ArcGIS 9.1, and arsenic concentrations were assigned to the cohort members’ addresses, with the spatial join functionality. First, the 170,403 (84%) of the cohort addresses located within one of the mapped water supply areas were assigned an estimated arsenic concentration, by the “point-in-polygon” procedure. This procedure allocates the attributes of the polygon to all points within it. Second, 28,355 (14%) of the addresses were assigned the arsenic concentrations of the nearest water utility, by application of the “point-to-point”-spatial join. By this procedure all points in one data set will be given the attributes of the points in another data set based on shortest distance. The last 3,581 (2%) of the cohort addresses had no geographic reference and were allocated a “missing value” as arsenic concentration.

### Arsenic exposure

We calculated two exposures for each cohort member. The first was a time-weighted average exposure, calculated as the arsenic concentration in drinking-water multiplied by the time lived at each address, summed for all residential addresses during the study period and divided by the total observation time, with the unit micrograms per liter (Equation 1).


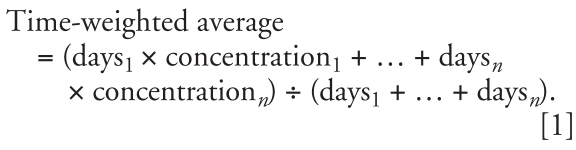


Second, we calculated the cumulated arsenic exposure by cumulating the products of “arsenic level × time” for each address occupied during the total observation period and multiplied by the total daily intake of tap water, with the unit milligram (Equation 2). The total daily intake of tap water was calculated as the sum of intake of tap water, coffee, tea, and fruit syrup diluted with tap water, which was reported at enrollment into the cohort.


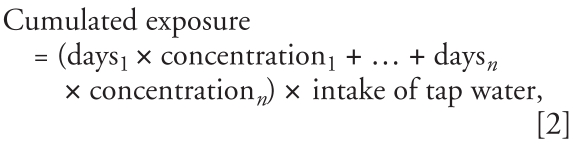


where *n* is the number of address periods.

### Potential confounding factors

We used data on smoking, alcohol consumption, education, body mass index, daily intake of fruit/vegetables, red meat, fat and dietary fibers, skin reaction to sun, hormone replacement therapy use, reproduction, and occupation collected at enrollment into the cohort to adjust for potential confounding factors for each type of cancer. Further, data was analyzed both with and without adjustment for enrollment area.

### Statistical analyses

Statistical analyses were carried out with the PHREG procedure of SAS 8.2 software (SAS Institute Inc., Cary, NC, USA). Cox proportional hazards models were used to estimate incidence rate ratios (IRRs) for cancer associated with the time-weighted average and cumulative arsenic exposure. Age was used as the time axis to ensure that the estimates were based on comparisons of individuals of the same age. The analyses were corrected for delayed entry, so that individuals were considered at risk only from the age at entry into the cohort. All analyses were stratified by sex. The age of 41 years was chosen as the starting point for calculating exposure because the oldest person in the cohort was 41 in 1970 when the residential histories began. This ensured that the exposure assessment started at the same age for all individuals. Both exposure measures—time-weighted average exposure per micrograms per liter since age 41 and total cumulated exposure per 5 mg since age 41—were included in the model as time-dependent variables. Cohort members were censored at age of death, emigration, cancer diagnosis or if they had moved to an address with unknown arsenic concentration.

The associations between arsenic exposure measures and cancer risk were modeled as straight lines, and the analyses were adjusted for known risk factors to control for potential confounding. The linear assumption was evaluated with a linear spline (Greenland 1995), with three boundaries placed at the quartiles of the time-weighted average exposure at the time of enrollment into the cohort. These were included as covariates in the Cox model, and linearity was assessed graphically and by a numerical test. To evaluate linearity, we also tested if an additional quadratic term improved the model fit. Further, we analyzed risk in association with quartiles of cumulated exposure at the time of diagnosis for cancer cases to investigate if the risk was higher or lower in association with the highest exposures.

Analyses were repeated using time-weighted average exposure to arsenic on the basis of addresses occupied from 1970 for all cohort members regardless of age. An additional analysis included a 5-year lag between exposure and cancer diagnosis, that is, excluding the 5 years before the cancer diagnosis in the exposure assessment of cases and a similar period for the non-cancer cohort members. We also repeated analyses after exclusion of individuals who had lived at an address for which the closest water utility was used as the expected source of drinking-water.

Arsenic concentrations were generally higher for persons enrolled in the Aarhus area than those in the Copenhagen area. This might imply confounding from risk factors that were not accounted for that differed between the Aarhus and the Copenhagen areas. Therefore, we repeated analyses with adjustment for enrollment area, knowing that such adjustment limited the exposure contrasts evaluated in the analyses.

For initial analyses (without adjustment for area) providing *p*-values ≤ 0.2, separate estimates of the association with arsenic exposure were calculated for the Aarhus and Copenhagen enrollment areas by including an interaction term in the model. This was to test the consistency of the results. A persistent association in both areas would strengthen the confidence in the result. We tested the null hypothesis that the effect of arsenic exposure was the same in the two areas (test for interaction).

On the basis of the results of previous studies, the associations between exposure to arsenic and cancers of the lung and bladder and non-melanoma skin cancer were estimated for never, former, and current smokers separately, and we tested for different effects of arsenic between the three smoking status categories.

## Results

Demographic, dietary, occupational, and other characteristics of the cohort members are presented in Table 1.

The time-weighted arsenic exposure of the cohort members calculated from 41 years of age up to date of enrollment varied between 0.05 and 25.3 μg/L, with a median concentration of 0.7 μg/L and a mean concentration of 1.2 μg/L. The exposure was generally higher among those enrolled in Aarhus than those enrolled in the Copenhagen area. Aarhus: mean = 2.3 μg/L, median = 2.1 μg/L; Copenhagen: mean = 0.7 μg/L, median = 0.6 μg/L (Table 2).

**Figure 3:**
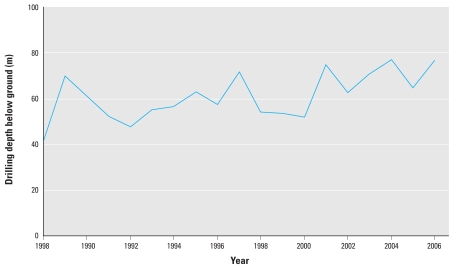
Groundwater drilling depths as a function of time, based on 3,396 measurements from drillings used for drinking-water.

Figure 3 illustrates a weak tendency towards increasing drilling depth over the last 18 years. Drilling depth explained 4% of the variation in arsenic concentration (*R*^2^ = 0.04; *n* = 3,396).

The results without adjustment for enrollment area (Table 3) showed no significant association between exposure to arsenic and risk for any type of cancer, except for non-melanoma skin cancer, for which higher arsenic exposure was associated with lower risk. The IRR for non-melanoma skin cancer was 0.88 [95% confidence interval (CI), 0.84–0.94] per micrograms per liter increase in time-weighted average exposure. A similar pattern was seen for cumulated arsenic exposure, with an IRR of 0.95 (95% CI, 0.92–0.97) for a 5-mg increase in exposure. The risk estimates for kidney cancer and melanoma were correspondingly low for both exposure measures but insignificant. Results adjusted for enrollment area (Table 3) showed virtually no effect for non-melanoma skin cancer, a stronger but still insignificant inverse risk association for melanoma skin cancer, and a significantly increased risk for breast cancer in association with time-weighted average exposure to arsenic (IRR = 1.05; 95% CI, 1.01–1.10).

Quartile-based analyses showed an IRR of 0.73 (95% CI, 0.59–0.91) for non-melanoma skin cancer for the upper quartile compared with the lower quartile of cumulated exposure from 41 years of age to date of diagnosis, but no decrease in risk was seen after adjustment for enrollment area (IRR = 1.14). The similar IRRs for melanoma skin cancer were 0.52 (95% CI, 0.28–0.98; *p* = 0.04) and 0.53 (95% CI, 0.32–0.88; *p* = 0.01) with and without adjustment for enrollment area respectively. The risk did not differ significantly between upper and lower quartile for any of the other cancers regardless of adjustment for enrollment area or not (all *p* > 0.12) (results not shown).

**Figure 4:**
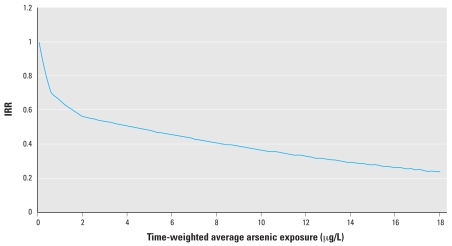
Dose–response curve for non-melanoma skin cancer. Reference, IRR = 1 at average time-weighted arsenic exposure of 0.05 μg/L.

Spline and quadratic tests showed deviation from a linear dose–response relation for cancers of the breast, lung, prostate, and liver, and for melanoma and non-melanoma skin cancers. When evaluated graphically, the dose–response relation for non-melanoma skin cancer showed a systematic nonlinear pattern, with a decreasing trend that leveled off with increasing exposure (Figure 4). The departure from linearity appeared to be random and nonbiological for the other cancers (results not shown).

In the overall analyses (Table 3, no adjustment for area), the risk estimates were affected to only a small extent by calculating time-weighted average exposure to arsenic from 1970 for all cohort members regardless of age, by introduction of a 5-year latency or by exclusion of individuals for whom the closest water utility was used as the expected source of drinking-water at the residence (results not shown). Furthermore, these results showed no significant interaction between arsenic and smoking, as the IRR estimates for never, former, and current smokers were not significantly different for cancers of the lung or bladder or non-melanoma skin cancer (all *p* > 0.12) (results not shown).

Table 4 shows inconsistent directions of the risk association in the two enrollment areas for non-melanoma skin cancer and a consistent direction of the risk association (inverse) for melanoma skin cancer, which was insignificant for both enrollment areas. Further, a consistent direction of the risk association for breast cancer (higher exposure was associated with higher risk) was observed, which was statistically significant in Aarhus when the time-weighted average exposure measure was applied (IRR = 1.06; 95% CI, 1.0–1.11; *p* = 0.02). None of the risk estimates differed significantly between the two areas (all *p* > 0.15).

## Discussion

We found no increased risk for cancers of the lung, bladder, kidney, liver, prostate, and colorectum, and for melanoma and non-melanoma skin cancers in association with low levels of exposure to arsenic through drinking-water. The risk for skin cancers decreased with increasing exposure. Results adjusted for enrollment area showed no significant risk associations except for with breast cancer, when the time-weighted average arsenic exposure was used and for melanoma skin cancer in the quartile-based analyses.

The median and mean arsenic exposure at enrollment were 0.7 and 1.2 μg/L, respectively, which are comparable to the concentrations found in Finland (median = 0.14 μg/L) (Kurttio et al. 1999), and the United States (mean = 2 μg/L) (Agency for Toxic Substances and Disease Registry 2005) but much lower than those found in some areas of Asia and Latin America.

Although previous studies provide evidence for an etiologic relationship between arsenic in drinking-water and cancer, they do not predict the cancer risk of low doses (Karagas et al. 2001). The arsenic levels in the Danish drinking-water are 100–1,000 times lower than those reported in studies from Asia and Latin America. It is possible that arsenic concentrations in the Danish drinking-water are below a low effect level; however, the results of the present study cannot rule out a weak adverse effect that is impossible to detect with the method used and the study size.

Conflicting results have been obtained in studies of arsenic and cancer conducted in areas of low arsenic concentrations in drinking-water. A Finnish case–cohort study reported increased risk for bladder cancer in association with exposure to arsenic (Kurttio et al. 1999) based on 61 cases and significant only for exposure 2–9 years before diagnosis for one of the three exposure measures used (Kurttio et al. 1999). Interpretation of the finding is therefore not straightforward. In contrast, our study, based on 214 cases, showed no increased bladder cancer risk. In line with the results of our study, the Finnish study did not find an association with kidney cancer (Kurttio et al. 1999). Studies carried out in the United States found no increased risk for bladder cancer with increasing arsenic exposure (Bates et al. 1995; Lamm et al. 2004; Steinmaus et al. 2003) in areas with arsenic concentrations in drinking-water of 0.5–160 μg/L. In one of these studies an insignificant tendency toward decreasing bladder cancer risk was seen with increasing exposure to arsenic ranging from 3 to 60 μg/L (Lamm et al. 2004). Another study in the United States showed an increased risk for prostate cancer in association with arsenic exposure (Lewis et al. 1999). We did not find such an association.

In the present study, higher exposure to arsenic was significantly associated with a lower risk for non-melanoma skin cancer in the overall analyses. Similar risk estimates were seen for melanoma skin cancer, although the results were not significant, possibly because of the small number of cases. These findings conflict with the results of some previous studies. In Taiwan, Wu et al. (1989) found a significant dose–response relation for non-melanoma skin cancer in association with exposure to arsenic, and a study in the United States showed a 1.9 times higher risk for skin cancer (type not specified) among persons exposed to drinking-water containing > 10 μg/L arsenic than those exposed to < 1.0 μg/L (Knobeloch et al. 2006). Another study of exposure to low levels of arsenic showed no association with non-melanoma skin cancer (Karagas et al. 2001). In a study of non-melanoma skin cancers in which arsenic in toenail tissue was used as bio-marker of exposure, a nonlinear dose–response relation was seen with low exposures, with an inverse association at low levels and an increasing risk with concentrations > 0.09–0.11 μg/g toenail, corresponding to 1–2 μg/L in drinking-water (Karagas et al. 2002). This result is consistent with our findings, as only a small proportion of the cohort members were exposed to drinking-water containing arsenic at > 2 μg/L.

In an experiment in cells *in vitro* a low dose of arsenic had a protective effect against oxidative stress and DNA damage, supporting the hypothesis that low doses of arsenic could protect against cancer. In this study, the point, at which the protective effect was out weighted by the toxic effect was 1 μmol/L corresponding to 50–60 μg/L (Snow et al. 2005). The findings of inverse risk associations for skin cancer in the present study further support the hypothesis that low doses of arsenic might be inversely associated with risk for skin cancer.

Nevertheless, the negative association between arsenic and non-melanoma skin cancer virtually disappeared when adjusted for enrollment area and when separate risk estimates were made for the two enrollment areas. This might be interpreted as confounding by some regional factor for which we did not adjust. For example, exposure to the sun is a risk factor for both melanoma and non-melanoma skin cancer (Scotto et al. 1996), and this might have confounded the results of the overall analysis if such exposure was more pronounced in the Copenhagen area, as the arsenic concentrations in drinking-water were generally higher in the Aarhus area. This interpretation is, however, contradicted by the fact that the inverse risk association for melanoma skin cancer persisted when risk estimates were calculated separately for the two enrollment areas. Further, the lower risk for confounding obtained by adjustment for enrollment area might be counterbalanced because this adjustment would make it more difficult to detect any effect of arsenic exposure, as part of the variation in exposure relates to differences between the two enrollment areas. Altogether, our finding of negative associations between arsenic and non-melanoma and melanoma skin cancers should be interpreted with caution.

To our knowledge, no epidemiologic study of an association between arsenic and cancer has included breast cancer. The borderline significance of the finding of an increased risk for breast cancer in association with arsenic exposure among cohort members enrolled in the Aarhus area should therefore be interpreted with caution, and more studies are needed to determine if arsenic in drinking-water is a risk factor for breast cancer.

Cases were identified in the virtually complete, reliable nationwide Danish Cancer Registry (Storm et al. 1997), and the Danish Population Registry provided complete follow-up of the cohort members. Although the exposure of the cohort members was assessed independently of who developed cancer, some degree of nondifferential misclassification of arsenic exposure inevitably occurred. This would in most cases be expected to bias risk estimates toward the neutral value (Rothman 2002), and it may therefore have contributed to the null results of the present study. Factors contributing to such exposure misclassification include the following: *a*) Recent arsenic measurement were assumed to represent historical exposure, in line with the approach of other studies (Bates et al. 2004; Kurttio et al. 1999). *b*) For 14% of the addresses, we assumed that the nearest water utility provided drinking-water to the address. However, exclusion of persons, who had lived at one of these addresses changed the risk estimates only marginally. *c*) Some water utilities might have closed during the study period, and supply structures might have changed. It is likely though that drinking-water from past and present water utilities that are spatially close would have similar arsenic concentrations, as the geologic composition of aquifers is fairly homogeneous over small geographic areas. *d*) There is a lack of information about exposure to arsenic through foodstuffs; however, arsenic in food occurs mainly in the less harmful organic form, and the typical Danish diet does not include arsenic-rich foods such as seaweed, skate, or stingray (Mohri et al. 1990). *e*) There is uncertainty in the reported intake of tap water. However, the results for time-weighted average arsenic exposure would not be affected by such misclassification, and the results for these two exposure measures gave similar results. *f*) Use of domestic water supply as a predictor for source of drinking-water implies some uncertainty (Jones et al. 2006). Because most of the water supply areas in the study covered large areas, such misclassification would apply mainly to persons, who traveled far between home and work.

Lack of information on residential histories before 1970 could also have led to misclassification of the exposure. Different migration patterns for cases and noncases before 1970 would imply differential misclassification, but we consider this unlikely because of the long time span between the period of unknown migrations (before 1970) and time of diagnosis for the cancer cases (after inclusion between 1994 and 1997). The strengths of our study include the large study population, the reliable population-based Danish registers, and adjustment for many potential confounding factors. Also, the precise link between place of residence and water supply and the measurements of arsenic concentrations in the drinking-water that was piped to the consumers adds strength to the study.

The limitations of the study include the overall low arsenic concentration in Danish drinking-water and lack of information on other sources of arsenic. Further, the exposure of cohort members before 1970 could not be estimated, as the residential histories before that date were unknown. Therefore we were not able to assess early-life arsenic exposure, which is an important limitation of this study because early environmental exposures might be most significant for cancer risk. Finally, measurement of arsenic in nails or urine would provide more precise estimates of the personal exposure and should be included in future studies whenever possible.

## Conclusion

We found no statistical significant association between arsenic concentrations in Danish drinking-water and the risk for cancers of the lung, bladder, kidney, liver, prostate, or colorectum. The results indicated inverse associations between arsenic concentrations in Danish drinking-water and risk for skin cancers, suggesting that arsenic might have a protective effect at low concentrations. The results also indicated that arsenic in drinking-water might increase the risk for breast cancer. The findings should be interpreted with caution, and more studies are needed to confirm the results.

## Figures and Tables

**Table 1:** Demographic, lifestyle, and dietary characteristics of the cohort.

Characteristic	No. (%)	Median (5th–95th percentiles)	Characteristic	No. (%)	Median (5th–95th percentiles)
Total population	56,378 (100)		Sun exposure		
Sex			Skin reaction to sun		
Male	26,876 (48)		Very sunburnt	3,592 (6)	
Female	29,502 (52)		Sunburnt	8,689 (15)	
Age at inclusion (years)		56 (50–64)	Blushing followed by tanning	32,025 (57)	
Smoking			Sun tanned, no other reaction	11,918 (21)	
Status			Missing data	154 (0)	
Never	19,739 (35)		Sun tan during summer		
Former	16,231 (29)		Very	12,639 (22)	
Current	20,373 (36)		Moderately	31,817 (56)	
Missing data	35 (0)		Little	10,940 (19)	
Duration (years)^a^		33 (6–46)	Not, only freckled	936 (2)	
Intensity (g tobacco/day)^a^		15 (4–35)	Missing data	46 (0)	
Alcohol			Hormone replacement therapy		
Status			Never	16,045 (54)	
Never	1,316 (2)		Previously	4,569 (15)	
Ever	55,062 (98)		Currently	8,852 (30)	
Intake (g/day)^b^		13 (1–65)	Missing data	36 (0)	
Education (years of school)			Years of use^c^		4 (0.5–18)
< 7	18,612 (33)		Births		
8–10	25,950 (46)		None	3,542 (12)	
> 10	11,787 (21)		Any	25,960 (88)	
Missing data	29 (0)		Number^d^		2 (1–4)
Body mass index (kg/m^2^)		26 (20–33)	Age at first^d^		23 (18–32)
Dietary intake					
Fruit and vegetables (g/day)		347 (108–803)	Ever occupied for at least 1 year in an industry or job associated with risk of developing:		
Fat (g/day)		81 (45–140)	Colorectal cancer^e^	2,612 (5)	
Dietary fiber (g/day)		20 (11–34)	Liver cancer^e^	2,612 (5)	
Red meat (g/day)		78 (32–166)	Kidney cancer^e^	2,612 (5)	
Tap water (L/day)		1.6 (0.7–2.9)	Lung cancer^f^	15,866 (28)	
			Breast cancer^g^	6,636 (12)	
			Bladder cancer^h^	13,261 (24)	
			Non-melanoma skin cancer^i^	697 (1)	

a Among former and current smokers.

b Among ever alcohol drinkers.

c Among current and former users.

d Among mothers.

e Waiter, cook.

f Mining, electroplating, manufacturing of shoes/leather products, metal processing (welding/painting), foundry/steel rolling mill, shipyard, glass industry, building industry (roof constructor/asphalt worker/demolition worker), truck/bus/taxi driver, manufacturing of asbestos/cement, asbestos insulation, cement article industry, china and pottery industry, butcher, painter, welder, auto mechanic, waiter, cook.

g Health care.

h Rubber industry, textile industry (dyeing), metal processing (painting), glass industry, truck/bus/taxi driver, painter, hairdresser, waiter, cook.

i Building industry (roof constructor/asphalt worker).

**Table 2:** Time-weighted average arsenic exposure from 41 years of age to date of enrollment.

	Arsenic concentration (μg/L)		
Percentile	Entire cohort (*n* = 56,378)	Enrolled in Copenhagen (*n* = 39,378)	Enrolled in Aarhus (*n* = 17,000)
Minimum	0.05	0.05	0.09
1st	0.05	0.05	0.4
5th	0.3	0.05	0.8
25th	0.6	0.5	2.0
50th	0.7	0.6	2.1
75th	2.0	0.9	2.1
95th	2.1	2.0	2.5
99th	5.7	2.0	18.1
Maximum	25.3	15.8	25.3

**Table 3:** Incidence rate ratios for cancer in association with arsenic exposure.

		Adjusted analysis				Further adjustment for area of enrollment		
Cancer	No. of cases	IRR	95% CI	*p*-Value	IRR	95% CI	*p*-Value
Time-weighted average exposure (μg/L)							
Colorectal^a^	441	0.97	0.90–1.05	0.49	0.93	0.84–1.04	0.21
Liver^b^	35	1.05	0.88–1.25	0.57	0.97	0.72–1.29	0.81
Lung^c^	402	0.99	0.92–1.07	0.78	0.99	0.90–1.08	0.76
Breast^d^	766	1.03	0.99–1.08	0.20	1.05	1.01–1.10	0.02
Prostate^e^	332	1.03	0.97–1.09	0.41	1.03	0.96–1.10	0.45
Kidney^f^	53	0.89	0.65–1.22	0.46	0.88	0.58–1.35	0.57
Bladder^g^	214	1.01	0.93–1.11	0.75	1.00	0.91–1.11	0.93
Melanoma skin^h^	147	0.89	0.73–1.07	0.20	0.80	0.59–1.08	0.14
Non-melanoma skin^i^	1,010	0.88	0.81–0.94	0.0004	0.99	0.94–1.06	0.85
Cumulated exposure (5 mg)							
Colorectal^a^	441	0.98	0.96–1.01	0.28	0.97	0.93–1.01	0.10
Liver^b^	35	0.99	0.89–1.10	0.79	0.89	0.73–1.08	0.24
Lung^c^	402	1.0	0.98–1.02	0.80	1.00	0.98–1.03	0.75
Breast^d^	766	1.0	0.99–1.02	0.61	1.01	0.99–1.03	0.21
Prostate^e^	332	1.0	0.99–1.03	0.44	1.01	0.99–1.03	0.44
Kidney^f^	53	0.94	0.84–1.06	0.33	0.94	0.81–1.09	0.38
Bladder^g^	214	1.0	0.98–1.04	0.55	1.01	0.98–1.04	0.69
Melanoma skin^h^	147	0.97	0.92–1.03	0.35	0.96	0.89–1.04	0.32
Non-melanoma skin^i^	1,010	0.95	0.92–0.97	< 0.0001	0.99	0.97–1.01	0.35

a Adjusted for smoking status, smoking duration, smoking intensity, education, body mass index (BMI), alcohol status, daily intake of alcohol, hormone replacement therapy (HRT) status, years of HRT use, occupation, daily intake of: red meat, dietary fibres, and fruits/vegetables.

b Adjusted for smoking status, smoking duration, smoking intensity, education, alcohol status, daily intake of alcohol, occupation.

c Adjusted for smoking status, smoking duration, smoking intensity, education, occupation, daily intake of fruits/vegetables.

d Adjusted for HRT status, years of HRT use, no. of births, age at first birth, education, alcohol status, daily intake of alcohol, daily intake of fruits/vegetables, BMI, occupation.

e Adjusted for education, BMI, daily intake of: fruits/vegetables and fat.

f Adjusted for smoking status, smoking duration, smoking intensity, education, BMI, occupation.

g Adjusted for smoking status, smoking duration, smoking intensity, education, occupation.

h Adjusted for education, skin reaction to sun, suntanned during summer.

i Adjusted for education, skin reaction to sun, suntanned during summer, occupation.

**Table 4:** Incidence rate ratios for cancer in association with arsenic exposure in the two enrollment areas.

			Time-weighted average exposure			Cumulated exposure		
Cancer	No. of cases	Area	IRR^a^	95% CI	*p-*Value	IRR^b^	95% CI	*p-*Value
Melanoma skin^c^	105	CPH	0.73	0.46–1.14	0.17	0.94	0.81–1.08	0.37
Melanoma skin^c^	42	ARH	0.85	0.61–1.20	0.36	0.97	0.90–1.05	0.47
Non-melanoma skin^d^	813	CPH	1.09	0.95–1.24	0.21	1.01	0.97–1.06	0.66
Non-melanoma skin^d^	197	ARH	0.97	0.90–1.05	0.46	0.98	0.95–1.01	0.22
Breast^e^	582	CPH	1.04	0.88–1.22	0.66	1.01	0.95–1.06	0.86
Breast^e^	184	ARH	1.06	1.01–1.11	0.02	1.01	0.99–1.03	0.20

Abbreviations: ARH, Aarhus enrollment area; CPH, Copenhagen enrollment area.

a Per μg/L time-weighted average arsenic exposure.

b Per 5 mg cumulated arsenic exposure.

c Adjusted for education, skin reaction to sun, sun tanned during summer.

d Adjusted for education, skin reaction to sun, sun tanned during summer, occupation.

e Adjusted for hormone replacement therapy status and years of use, no. of births, age at first birth, education, alcohol status, daily intake of alcohol, daily intake of fruits/vegetables, body mass index, occupation.

## References

[b1-ehp0116-000231] Abernathy CO, Chappell WR, Meek ME, Gibb H, Guo HR (1996). Is ingested inorganic arsenic a “threshold” carcinogen?. Fundam Appl Toxicol.

[b2-ehp0116-000231] Abernathy CO, Thomas DJ, Calderon RL (2003). Health effects and risk assessment of arsenic. J Nutr.

[b3-ehp0116-000231] Agency for Toxic Substances and Disease Registry (2005). Toxicological Profile for Arsenic (Draft for Public Comment).

[b4-ehp0116-000231] Bates MN, Rey OA, Biggs ML, Hopenhayn C, Moore LE, Kalman D (2004). Case-control study of bladder cancer and exposure to arsenic in Argentina. Am J Epidemiol.

[b5-ehp0116-000231] Bates MN, Smith AH, Cantor KP (1995). Case-control study of bladder cancer and arsenic in drinking water. Am J Epidemiol.

[b6-ehp0116-000231] Burns FJ, Uddin AN, Wu F, Nadas A, Rossman TG (2004). Arsenic-induced enhancement of ultraviolet radiation carcinogenesis in mouse skin: a dose-response study. Environ Health Perspect.

[b7-ehp0116-000231] Chen CJ, Chuang YC, You SL, Lin TM, Wu HY (1986). A retrospective study on malignant neoplasms of bladder, lung and liver in blackfoot disease endemic area in Taiwan. Br J Cancer.

[b8-ehp0116-000231] Chen CJ, Kuo TL, Wu MM (1988). Arsenic and cancers. Lancet.

[b9-ehp0116-000231] Cohen SM, Ohnishi T, Arnold LL, Le XC (2007). Arsenic-induced bladder cancer in an animal model. Toxicol Appl Pharmacol.

[b10-ehp0116-000231] Farago ME, Thornton I, Kavanagh P, Elliott P, Leonardi GS, Abernathy CO, Calderon RL, Chappell WR (1997). Health aspects of human exposure to high arsenic concentrations in soil in south-west England. Arsenic: Exposure and Health Effects.

[b11-ehp0116-000231] Ferreccio C, Gonzalez C, Milosavjlevic V, Marshall G, Sancha AM, Smith AH (2000). Lung cancer and arsenic concentrations in drinking water in Chile. Epidemiology.

[b12-ehp0116-000231] Greenland S (1995). Dose-response and trend analysis in epidemiology: alternatives to categorical analysis. Epidemiology.

[b13-ehp0116-000231] Hopenhayn-Rich C, Biggs ML, Fuchs A, Bergoglio R, Tello EE, Nicolli H (1996). Bladder cancer mortality associated with arsenic in drinking water in Argentina. Epidemiology.

[b14-ehp0116-000231] Hopenhayn-Rich C, Biggs ML, Smith AH (1998). Lung and kidney cancer mortality associated with arsenic in drinking water in Cordoba, Argentina. Int J Epidemiol.

[b15-ehp0116-000231] Jones AQ, Dewey CE, Dore K, Majowicz SE, McEwen SA, Waltner-Toews D (2006). Exposure assessment in investigations of waterborne illness: a quantitative estimate of measurement error. Epidemiol Perspect Innov.

[b16-ehp0116-000231] Karagas MR, Stukel TA, Morris JS, Tosteson TD, Weiss JE, Spencer SK (2001). Skin cancer risk in relation to toe-nail arsenic concentrations in a US population-based case-control study. Am J Epidemiol.

[b17-ehp0116-000231] Karagas MR, Stukel TA, Tosteson TD (2002). Assessment of cancer risk and environmental levels of arsenic in New Hampshire. Int J Hyg Environ Health.

[b18-ehp0116-000231] Knobeloch LM, Zierold KM, Anderson HA (2006). Association of arsenic-contaminated drinking-water with prevalence of skin cancer in Wisconsin’s Fox River Valley. J Health Popul Nutr.

[b19-ehp0116-000231] Kurttio P, Pukkala E, Kahelin H, Auvinen A, Pekkanen J (1999). Arsenic concentrations in well water and risk of bladder and kidney cancer in Finland. Environ Health Perspect.

[b20-ehp0116-000231] Lamm SH, Engel A, Kruse MB, Feinleib M, Byrd DM, Lai S (2004). Arsenic in drinking water and bladder cancer mortality in the United States: an analysis based on 133 U.S. counties and 30 years of observation. J Occup Environ Med.

[b21-ehp0116-000231] Lewis DR, Southwick JW, Ouellet-Hellstrom R, Rench J, Calderon RL (1999). Drinking water arsenic in Utah: a cohort mortality study. Environ Health Perspect.

[b22-ehp0116-000231] Marshall G, Ferreccio C, Yuan Y, Bates MN, Steinmaus C, Selvin S (2007). Fifty-year study of lung and bladder cancer mortality in Chile related to arsenic in drinking water. J Natl Cancer Inst.

[b23-ehp0116-000231] Mohri T, Hisanaga A, Ishinishi N (1990). Arsenic intake and excretion by Japanese adults: a 7-day duplicate diet study. Food Chem Toxicol.

[b24-ehp0116-000231] Rossman TG, Uddin AN, Burns FJ (2004). Evidence that arsenite acts as a cocarcinogen in skin cancer. Toxicol Appl Pharmacol.

[b25-ehp0116-000231] Rothman K (2002). Biases in study design. Epidemiology: An Introduction.

[b26-ehp0116-000231] Schoen A, Beck B, Sharma R, Dube E (2004). Arsenic toxicity at low doses: epidemiological and mode of action considerations. Toxicol Appl Pharmacol.

[b27-ehp0116-000231] Scotto J, Fears TR, Fraumeni J, Schottenfield D, Fraumeni J (1996). Solar radiation. Cancer Epidemiology and Prevention.

[b28-ehp0116-000231] Smedley P, Kinniburgh DG, Selinus O, Alloway BJ, Centeno JA, Finkelman RB, Fuge R, Lindh U (2005). Arsenic in groundwater and the environment. Essentials of Medical Geology: Impacts of the Natural Environment on Public Health.

[b29-ehp0116-000231] Snow ET, Sykora P, Durham TR, Klein CB (2005). Arsenic, mode of action at biologically plausible low doses: What are the implications for low dose cancer risk?. Toxicol Appl Pharmacol.

[b30-ehp0116-000231] Steinmaus C, Yuan Y, Bates MN, Smith AH (2003). Case-control study of bladder cancer and drinking water arsenic in the western United States. Am J Epidemiol.

[b31-ehp0116-000231] Storm HH, Michelsen EV, Clemmensen IH, Pihl J (1997). The Danish Cancer Registry—history, content, quality and use. Dan Med Bull.

[b32-ehp0116-000231] Tchounwou PB, Centeno JA, Patlolla AK (2004). Arsenic toxicity, mutagenesis, and carcinogenesis—a health risk assessment and management approach. Mol Cell Biochem.

[b33-ehp0116-000231] Thomsen R, Søndergaard VH, Sørensen KI (2004). Hydrogeological mapping as a basis for establishing site-specific ground-water protection zones in Denmark. Hydrogeol J.

[b34-ehp0116-000231] Tjønneland A, Olsen A, Boll K, Stripp C, Christensen J, Engholm G (2007). Study design, exposure variables, and socioeconomic determinants of participation in Diet, Cancer and Health: a population-based prospective cohort study of 57,053 men and women in Denmark. Scand J Public Health.

[b35-ehp0116-000231] Tsuda T, Babazono A, Yamamoto E, Kurumatani N, Mino Y, Ogawa T (1995). Ingested arsenic and internal cancer: a historical cohort study followed for 33 years. Am J Epidemiol.

[b36-ehp0116-000231] Vahter M, Li L, Nermell B, Rahman A, El Arifeen S, Rahman M (2006). Arsenic, a global public health problem [Abstract]. Toxicol Lett.

[b37-ehp0116-000231] Waalkes MP, Liu J, Diwan BA (2007). Transplacental arsenic carcinogenesis in mice. Toxicol Appl Pharmacol.

[b38-ehp0116-000231] Wanibuchi H, Salim EI, Kinoshita A, Shen J, Wei M, Morimura K (2004). Understanding arsenic carcinogenicity by the use of animal models. Toxicol Appl Pharmacol.

[b39-ehp0116-000231] Wu MM, Kuo TL, Hwang YH, Chen CJ (1989). Dose-response relation between arsenic concentration in well water and mortality from cancers and vascular diseases. Am J Epidemiol.

